# Type 2 diabetes mellitus in the pathophysiology of Alzheimer's disease

**DOI:** 10.1590/1980-57642016dn11-020002

**Published:** 2017

**Authors:** Aparecida Marcelino de Nazareth

**Affiliations:** 1 Physiotherapist, Specialist in Neurofunctional Physical Therapy, Master of Neurosciences from the (UFSC), SC, Brazil, and PhD in Sciences (Pharmacology and Medicinal Chemistry) from the Federal University of Rio de Janeiro (UFRJ), RJ, Brazil

**Keywords:** Alzheimer's disease, type 2 diabetes mellitus, inflammation, doença de Alzheimer, diabetes mellitus tipo 2, inflamação

## Abstract

Both Alzheimer's disease (AD) and type 2 diabetes mellitus (DM) are two common forms of disease worldwide and many studies indicate that people with diabetes, especially DM, are at higher risk of developing AD. AD is characterized by progressive cognitive decline and accumulation of β-amyloid (Aβ) forming senile plaques. DM is a metabolic disorder characterized by hyperglycemia in the context of insulin resistance and relative lack of insulin. Both diseases also share common characteristics such as loss of cognitive function and inflammation. Inflammation resulting from Aβ further induces production of Aβ_1-42_ peptides. Inflammation due to overnutrition induces insulin resistance and consequently DM. Memory deficit and a decrease in GLUT4 and hippocampal insulin signaling have been observed in animal models of insulin resistance. The objective of this review was to show the shared characteristics of AD and DM.

## Abstract (pt)

Tanto a doença de Alzheimer (DA) e diabetes mellitus tipo 2 (DM2) são duas formas comuns de doenças no mundo e muitos estudos indicam que pessoas com diabetes, especialmente DM2, estão em maior risco de desenvolver DA. DA é caracterizada por um declínio cognitivo progressivo e acúmulo de β-amilóide (Aβ) formando placas senis. A DM2 é um distúrbio metabólico caracterizado por hiperglicemia no âmbito da resistência à insulina e falta relativa de insulina. Ambas as doenças também compartilham características comuns tais como a perda da função cognitiva e inflamação. A inflamação resultante de Aβ induz ainda mais a produção de peptídeos Aβ_1-42_. A inflamação devido à hipernutrição induz resistência a insulina e consequentemente T2D. Um deficit de memória e uma diminuição de sinalização de insulina hipocampal e GLUT4 foram observados em modelos animais de resistência a insulina. O objetivo desta revisão é mostrar as características comuns das DA e DM2.

## INTRODUCTION

Many researchers have struggled to understand the molecular basis of the pathophysiology of Alzheimer's disease (AD) because an exponential number of cases have been predicted for the coming decades and more effective treatments will be required to prevent or halt progression of the disease.

AD is characterized by loss of cognitive function evolving to dementia and death. Despite decades of research, the etiology of the disease is still poorly understood and data have shown that type 2 diabetes mellitus (DM) is a risk factor for AD.^1-4^ Moreover, it has been reported that metabolic disorders resulting from a high-fat diet and obesity, which can develop to and from DM, result in cognitive decline and AD-like dementia.^2,5^ Furthermore, DM can lead to increased immune system activity and a consequent increase in the secretion of proinflammatory cytokines, which can contribute to brain neuroinflammation. Neuroinflammation is one of the pathophysiological features of AD.^6,7^ Numerous studies have proposed that inflammatory dysfunctions are associated with neurodegenerative disorders in both animal models and humans. Moreover, AD brains exhibit defective insulin signaling, and, more importantly, decreased responsiveness to insulin.^8^ Relative insulin deficiency and insulin resistance are characteristics of DM.^9,10^ The aim of this review was to show the shared characteristics of AD and DM, for example, insulin resistance and inflammation.

## ALZHEIMER'S DISEASE

Alzheimer's disease (AD) has been studied for decades. First described in 1907 by the German physician, Alois Alzheimer,^11^ it is a progressive neurodegenerative disorder^12^ characterized by β-amyloid plaques and tangles of hyperphosphorylated tau proteins, besides cholinergic dysfunction. There is a sporadic form of the disease^13,14^ and average survival is about eight years.^12^

The typical AD symptomatology is severe and progressive impairment of cognitive function,^12^ which includes memory loss and language problems^15^ as well as non-cognitive dysfunction (executive) often followed by behavioral disorders such as agitation, aggressiveness and depression^12,15^ (Table 1).

**Table 1:** Diagnostic criteria for Alzheimer's disease.

Symptoms (Initial phase)	Memory: difficulty remembering recent events.
	Language: difficulty finding commonly used words.
	Performance on ADLs*: difficulty in tasks requiring multiple steps (e.g., preparing dinner).
	Spatial orientation: difficulty in orienting for familiar routes (returning home).
	Behavior: apathy, depression, agitation and aggression.
Laboratory tests	Mini-Mental State Examination (MMSE), computed tomography, magnetic resonance imaging, or positron emission tomography, to rule out other possible causes for symptoms.
	Differential diagnosis: Pick's disease; Vascular dementia; Dementia of Lewy Bodies and others.

* Activities of daily living (ADLs): personal tasks or skills pertaining to daily life.

Neocortex and hippocampus seem to be the areas most affected by specificity of the disease^12^ and the loss of neurons in these areas is responsible for their atrophy, which is inherent to cognitive dysfunction, especially of memory, and the disease diagnosis.^16-18^ Apart from these areas, the subcortical nuclei that connect the cortex are also affected, including the cholinergic nucleus basalis of Meynert and medial septum.^12,19^

**Figure 1:**
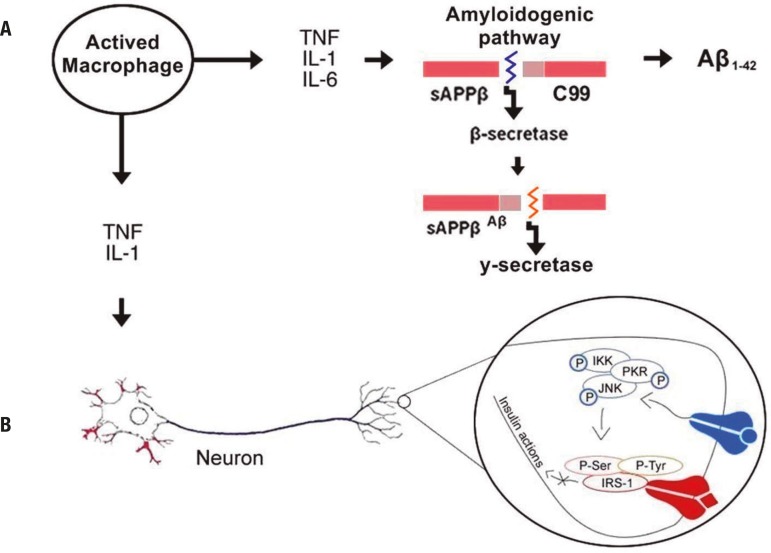
Inflammatory mediators contribute to increased β-amyloid production and reduced insulin activity. [A] Cytokines released by activated resident macrophages of the CNS (microglia) stimulates the amyloidogenic pathways of APP promoting an increase in β-amyloid levels in AD. Accumulation of Aβ together with inflammation induces oxidative stress and decreases brain insulin signaling^8^. [B] High accumulation of glucose and fat in peripheral blood induces the activation of immune cells such as macrophages. The proinflammatory cytokines released by these cells bind to their receptor located in neurons and promote the activation of molecules such as JNK leading to serine phosphorylation of the IRS-1 receptor, thereby triggering insulin resistance and blocking of insulin action in neuron cells. APP: amyloid precursor protein; Aβ: β-amyloid; C99: Protein C99; TNF: Tumour necrosis factor; IL-1, IL-6: Interleukins 1 and 6; IKK: I kappa B kinase; PKR: RNA-activated protein kinase; JNK: c-Jun N-terminal kinases; Ser: Serine; Tyr: Tyrosine; IRS-1: Insulin receptor substrate 1; • in blue: TNF bound in TNF receptor; ♦ in red: Insulin bound in its receptor.

β-amyloid protein is found diffusely in the brain of Alzheimer patients.^20^ It should be highlighted that the disease onset occurs due to the accumulation of this protein, which leads to neuronal dysfunction and death.^21-23^ β-amyloids are peptides of 39-43 amino acid residues^24^ and, although produced by nearly all cells, there are no reports about their function.^25^ It is derived from the amyloid precursor protein (APP),^26^ which is cleaved in two pathways. One of these occurs by the action of α- and γ-secretase enzymes and is non-amyloidogenic. This path gives rise to a protein called sAPPα, which is soluble and seems to be involved in neuroprotection.^27,28^ The other is the amyloidogenic path (Figure 1). In this pathway, APP cleaved by β-secretase generates sAPPβ and a membrane-bound C-terminal fragment (C99), which, subsequently cleaved by γ-secretase produces the Aβ peptide.^28,29^ Some reports state that one way of preventing βA formation is by inhibiting these enzymes.^21^

APP, whose physiological function is yet not clearly elucidated,^30^ has extracellular, membrane and cytoplasmic portions. β-amyloid derives from the membrane portion and is found in both soluble (monomeric and dimeric) and insoluble (aggregate) forms in interstitial and cerebrospinal fluids (CSF).^26^ The most common isoforms of β-amyloid are Aβ_1-40_ and Aβ_1-42_. This latter isoform has a hydrophobic nature and aggregates faster than Aβ_1-40._^31^ The Aβ deposition formation induces microglia and astrocyte activation, promoting the onset of neuroinflammation.^6,7^

Neuroinflammation is another feature that seems to be involved in the pathophysiology of AD. The presence of microglia and astrocytes around β-amyloid is associated with supra-regulation of pro-inflammatory cytokines, such as IL-1, IL-6 and TNF-α,^32^ which mediate the stimulation of APP production and the amyloidogenic pathway, inducing the production of Aβ_1-42_ peptides^33^ (Figure 1A). All these factors, the activation of the innate immune response, and the production of Aβ collaborate toward the dysfunction and death of neurons. It is known that the accumulation of Aβ together with inflammation induces oxidative stress and decreases brain insulin signaling.^8^ One of the functions of insulin is the regulation of key processes for learning such as synaptic density, dendritic plasticity and the promotion of neuronal survival.^34^

According to some reports, the interaction of environmental and genetic factors may trigger AD.^13^ With regard to genetic factors, data indicate that the incidence of this disease appears to be initiated by the mutation of APP genes and presenilin proteins 1 and 2 (PSEN 1 and PSEN 2),^21^ which are also involved in β-amyloid^13^ formation.

In addition to APP and PSEN gene mutations, positional cloning has revealed that the apolipoprotein E (APOE) is also involved in the onset of the disease.^35^ APOE is a 299 amino acid lipoprotein encoded by the APOE gene.^36,37^ Human ApoE is synthesized in various organs, especially the liver and brain, playing a key role in lipid metabolism,^36^ and can increase β-amyloid peptide aggregation in the brain.^38^

Besides extracellular deposits of β-amyloid, in AD there is formation of intracellular neurofibrillary tangles of hyperphosphorylated tau protein and consequent loss of neuronal synapses.^12^ Tau is a family of phosphoproteins^39^ associated with microtubules, particularly in neurons^40^ located mainly in the axonal cone and axonal endings of these cells.^39^

Phosphorylation of Tau protein is regulated from fetal to adult life.^41^ However, in AD, tau's microtubule binding domain is hyperphosphorylated. Tau hyperphosphorylation decreases its affinity for microtubules^42^ promoting neuronal dysfunction due to loss of normal cell morphology, axonal transport, synaptic dysfunction and neurodegeneration.^43^ High levels of abnormal hyperphosphorylated tau protein are observed in Alzheimer patient neuronal cytosol^44^ and CSF.^45^ Evidence indicates that this hyperphosphorylation is induced by β-amyloid.^46^

In addition, it has been reported that the hyperphosphorylation of Tau protein is associated with an increase in cytokine levels.^34,47^ Li et al. (2003), upon placing microglia previously activated with Aβ in co-culture with neurons, observed an increase in Tau phosphorylation as well as a decrease in the synaptophysin levels of these cells. These same effects occurred after treatment of neuronal cells with IL-1β. In contrast, there was an attenuation of these effects by treatment with the IL-1β receptor antagonist (IL-1ra), and likewise with an anti-IL-1 antibody.^47^

Moreover, other authors have shown that the application of IL-6 directly to rat hippocampal neurons promoted hyperphosphorylation of Tau protein and that this effect was dependent on cdk5/p35 complex, one of the main kinases implicated in tau hyperphosphorylation in neurodegenerative diseases.^48^

It is well established that there is degeneration of cholinergic neurons in Alzheimer's patients.^12^ The remotest AD hypothesis is founded on cholinergic dysfunction.^19^ Currently, the available drug therapies are based on this cholinergic hypothesis^49^ and there are several therapeutic interventions aimed at improving cholinergic transmission in these patients.^19,49^

The neurotransmission promoted by the acetylcholine neurotransmitter, among other functions, is involved in cognition, particularly memory.^19^ However, the role of the cholinergic system in the pathogenesis of AD remains unknown.^50^ There are indications, though, that picomolar concentrations of β-amyloid induce a blockade of choline uptake as well as the release of choline from the cell, thus promoting the dysfunction of the cholinergic system.^20^ Furthermore, there is degeneration of cholinergic neurons, especially in the basal nucleus of Meynert, in this disease.^12,19^

Studies show a reduction in acetylcholinesterase and choline acetyltransferase (ChAT) activity in the brain of Alzheimer's patients relative to the brains of normal individuals.^51^ Furthermore, there are reports of a reduction in ChAT activity in human postmortem cerebral cortex^52^ and of a correlation between cognitive impairment of Alzheimer's patients and both ChAT activity and acetylcholine synthesis.^12^ Also, a reduction in the levels of muscarinic and nicotinic acetylcholine receptors, as well as presynaptic markers of cholinergic neurons in postmortem brains of patients with the disease, was observed. The cholinergic deficit in Alzheimer's disease has been attributed to the neuronal loss resulting from deposition of Aβ.^53^

## TYPE 2 DIABETES MELLITUS

DM is implicated as one of the risk factors for AD,^1,3^ and data suggest that Alzheimer's patients have a high risk of developing type 2 diabetes.^54^ There appears to be a bidirectional relationship between the two diseases.

Diabetes mellitus is a group of metabolic diseases characterized by chronic hyperglycemia resulting from defects in insulin secretion or action. Types of diabetes include type 1 diabetes mellitus (or insulin-dependent diabetes) that derives from the autoimmune destruction of pancreatic β cells, responsible for the production of insulin,^55,56^ and type 2 diabetes (or insulin-independent diabetes),^57^ characterized by high blood glucose levels in the context of relative insulin deficiency and insulin resistance^9,10^ (Table 2).

**Table 2:** Simplified diagnostic criteria for type 2 diabetes mellitus (DM).

Symptoms	Laboratory tests		
	**Glycemia after 2 h**	Glycemia after fasting*	**Glycated HbA1C**
Presence/absence of polyuria, polydipsia and polyphagia	Normal: < 140 mg/dl	Normal: < 110 mg/dl	Normal: < 6%
	GT**: ≥ 140 mg/dl	GT: ≤ 126 mg/dl	GT: 6.0-6.4%
	DM: ≥ 200 mg/dl	DM: ≥ 126 mg/dl	DM: ≥ 6.5%

* Fasting period comprises no caloric intake for at least 8 hours.

** The test of tolerance to glucose is performed if there are no associated symptoms. This test consists of fasting blood collection, followed by ingestion of 75 g of glucose and then repeat blood collection after two hours. DM: Diabetes Mellitus; GT: Glucose Tolerance; Hb: hemoglobin.

Insulin is a peptide hormone consisting of 51 amino acids that metabolizes glucose and promotes its uptake by cells.^10,58,59^ Insulin is also responsible for the anabolism of carbohydrates, proteins and lipids. Its deficiency may generate metabolic abnormalities of these molecules.^58,60^

Insulin promotes its effect by binding to its receptors (IR), which belong to the tyrosine kinase receptor class. In the intramembrane, tyrosine kinase domains are bound to the insulin receptor substrate 1 and 2 (IRS-1 and IRS-2).^10,58^ These mediate the response to insulin via the serine/threonine kinase family known as Protein kinase B (PKB, also known as Akt) and protein kinase C (PKC), which phosphorylate several residues of IRS serine/threonine involved in the metabolic insulin response.^58,60^ Akt and PKC kinases are essential in the development of diabetes and are associated with hyper-insulinemia, dyslipidemia, and insulin resistance.^59^ Also, other non-insulin-dependent kinases can phosphorylate both insulin-dependent substrates. These kinases include protein kinase activated by cAMP (PKA), protein kinase c-Jun N-terminal (JNK) and kinase 2 of the G protein-coupled receptor kinase 2 (GRK2).^60^ Insulin receptors are densely expressed in the hypothalamus, where they have a role in the regulation of body weight and feeding behavior, as well as in the cerebral cortex, entorhinal cortex and hippocampus, an area involved with memory.^61^

Insulin also promotes its effect by binding itself to insulin-like growth factor 1 (IGF-1R).^10^ This factor is a hormone produced mainly in the liver and central nervous systems (CNS) that acts as a neurotrophic peptide; it can promote synaptic plasticity through the activation of the IRS-1 signaling pathway phosphatidylinositol 3-kinase (PI3K) and Akt.^62,63^ IRS-2 receptor also seems to be involved with neuroplasticity processes such as learning and memory.^64^ Insulin improves cognitive performance in humans and animals.^4^

Both neurons and glial cells express IGF-1R and IR. However, it has been suggested that neurons synthesize IGF-1 under physiological conditions, whereas astrocytes are produced after injuries.^65^ With regard to insulin, its synthesis occurs only in neurons and not in glial cells.^10,66^

Besides insulin and IGF-1, there is the Glucagon-like peptide 1 (GLP-1) in the periphery and CNS. This is an insulinotropic hormone with neurotransmitter activity whose properties and functions are similar to insulin and IGF-1. GLP-1 is secreted by intestinal cells and neurons, and its receptor, GLP-1R, is widely expressed in the brain, including the cerebral cortex and hippocampus.^67,68^ It has been suggested that glial cells express GLP-1R and its ligand^69^ only in pathological conditions, such as neurodegenerative pathologies.

The main feature of the pathogenesis of DM is insulin resistance.^70^ Resistance to insulin appears to occur via genetic factors and/or failure in the recognition of the hormone by IR due to an increase in the levels of fatty acids, glycerol and glucose.^71^ It has been shown that high accumulation of glucose and fat in the blood induces the activation of immune cells, and thereby the secretion of proinflammatory cytokines, such as IL-1β and TNF-α. These cytokines promote the activation, in neurons, of molecules such as JNK leading to serine phosphorylation of the IRS-1 receptor, inhibiting tyrosine phosphorylation and triggering insulin resistance (Figure 1B).^72,73^

IL-1β in β cells of pancreatic islets and TNF-α is activated through several transcriptional pathways such as NF-κB, caspases and inflammasomes. Once activated, they bind to their respective receptors and recruit several other proinflammatory mediators. The increase in proinflammatory cytokines induces a chronic inflammatory process and reactive oxygen species (ROS) production.^72,73^ Oxidative stress promotes the activation of molecules such as JNK and thus insulin resistance.^74^ Insulin resistance and chronic peripheral hyperinsulinemia implies down-regulation of insulin transport to the brain, possibly inducing insulin deficiency in it.^75^

## TYPE 2 DIABETES MELLITUS IN ALZHEIMER'S DISEASE

Research has shown that DM may develop due to a high fat diet and obesity. Also, some studies have indicated that chronic ingestion of high-fat diets and DM are some of the risk factors for decline in cognitive function and for a dementia similar to AD.^5,76,77^ In a study using magnetic resonance imaging, DM patients showed reduced hippocampal volume and accelerated cognitive decline compared with healthy elderly individuals.^78^

In addition, another study showed, using a Senescence-accelerated mouse prone model (SAMP8), that animals subjected to experimental induction of type

2 diabetes exhibited memory deficit compared to nondiabetic SAMP8 mice. This memory deficit was observed using the Morris water maze.^79^ The authors also showed that these animals exhibited increased β-amyloid proteins in the brain and hyperphosphorylated Tau proteins in the hippocampus, indicating changes similar to those observed in Alzheimer's dementia.

Winocur et al. (2005) showed memory deficit and decrease in expression of insulin-dependent glucose transporter (GLUT4) and hippocampal insulin signaling in an animal model of insulin resistance and obesity.^4^ The authors pointed out that a deficit in insulin signaling may contribute to injuries in peripheral tissues of diabetes patients and the same can occur with hippocampal tissue inducing a deficit in memory in these animals. Also, other researchers showed that decreased sensitivity to insulin has been associated with reduced verbal fluency and with cortical volume reduction of temporal lobes in healthy elderly individuals.^80^

AD transgenic models submitted to a high-fat diet showed increases in β-amyloid and tau protein as well as activated astrocytes in mice brain cortex.^81^ Senescence-accelerated mice treated with a high-fat diet also showed an increase in β-amyloid and tau protein levels.^79^ By contrast, calorie restriction has been shown to reduce the deposition of β-amyloid in both elderly mice^82^ and transgenic models of AD.^83^ These data suggest a close relationship between type 2 diabetes and AD.

**Figure 2:**
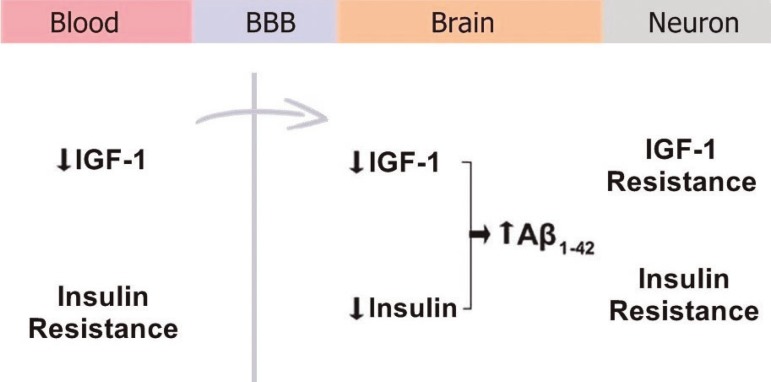
Uptake of IGF-1 and insulin into the brain. A reduction of IGF-1 and peripheral insulin resistance promotes a reduction in uptake of IGF-1 and insulin into the brain, resulting in accumulation of Aβ. Neuronal insulin resistance contributes to Aβ accumulation because the insulin degrading enzyme (IDE) also degrades Aβ. BBB: Blood-brain barrier; IGF-1: Insulin-like growth factor-1; Aβ: β-amyloid.

In addition, another study indicated increased secretion of Aβ by glucose and insulin in vitro and that Aβ was produced by adipose tissue cells in a concentration similar to that produced in vivo.^84^ The authors suggested that adipocytes treated with Aβ reduced IRS-2 expression, which is involved with memory, and phosphorylation of Akt-1. A reduction of IRS-2 activation in brains of Alzheimer patients was associated with IGF-1 resistance. Moreover, a reduction in IGF-1 and peripheral insulin resistance leads to reduced uptake of IGF-1 and insulin into the brain, resulting in accumulation of Aβ^85,86^ (Figure 2). Neuronal insulin resistance contributes to Aβ accumulation because the insulin-degrading enzyme (IDE) also degrades Aβ. The accumulation of β-amyloid promotes resistance to IGF-1 and insulin resistance. In contrast, a reduction in Aβ occurs among rodents with elevated levels of IGF-1.^87^ This indicates a role of insulin signaling in the deposition of Aβ in the brain.

Type 2 diabetes is characterized by hyperglycemia, decreased production of insulin or its availability, and insulin resistance.^57^ In this context, intraperitoneal injections of Aβ_1-42_ promoted hyperglycemia and insulin resistance in vivo via JAK2 in mice.^88^ In the study, insulin resistance appeared to be mediated by the activation of JNK, which induced inhibition of insulin signaling.^89^ This data was obtained by intracerebroventricular injection of Aβ oligomers in non-human primates and through in vitro hippocampal neuron investigation.

Studies indicate a reduction in glucose metabolism as well as changes in brain energy metabolism and impaired neuronal insulin signaling in AD patients.^14,90^ Insulin signaling, as measured by phosphorylation of AkT, was also found to be impaired by Aβ_1-42_ injection in rat hippocampus^91^ and in cultured hippocampal neurons by physiological inhibition of IRS-1pTyr.^89^ Moreover, hyperinsulinemia also promotes Aβ_1-42_ increase in the CSF of normal elderly.^92^ However, the mechanisms by which type 2 diabetes and AD interaction occurs remain unknown.

**Figure 3:**
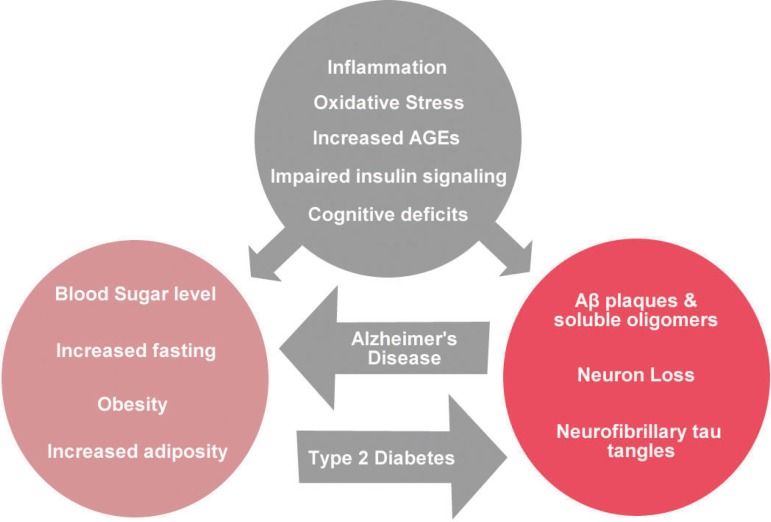
Distinct and shared symptoms of Type 2 Diabetes and Alzheimer's disease. Metabolic changes that occur in type 2 diabetes mellitus, such as hyperglycemia and obesity, can induce the presence of Aβ plaques, hyperphosphorylated Tau protein and loss of neurons. Aβ plaques can induce insulin resistance. The pathophysiology of both pathologies includes inflammation, increased oxidative stress, cognitive deficit and insulin resistance.

In both conditions, type 2 diabetes and AD, increased oxidative stress^93,94^ and chronic inflammation^57,94^ occur (Figure 3). The response to injury in the peripheral nervous system (PNS) and CNS by the activation of microglia and astrocytes is a normal and beneficial response. Nevertheless, an intense inflammatory response may promote the production of excess cytokines and oxidative stress leading to cell death.^94^ Neuroinflammation aggravates insulin resistance through the inhibition of IR signaling by the activation of the TNF-α receptor (TNFR).^95^ The binding of insulin to its receptor induces tyrosine phosphorylation of IRS, initiating intracellular signaling of insulin. When activated, TNFR activates the JNK pathway, one of which blocks insulin signaling by serine phosphorylation of IRS-1.^96^ This infers an insulin signaling deficit in CNS and PNS.

Cytokines and chemokines produced by both adipose tissue resident macrophages and by adipocytes of obese patients can cross the blood-brain barrier and contribute to the onset of brain inflammation.^97,98^ Also, change in the immune system accompanies physiological aging (inflamm-aging).^99^ In this context, it has been suggested that peripheral inflammatory mediators (TNF-α, IL6, IL-1β) due to inflammation and/or infection, or metabolic disorders related to obesity, including type 2 diabetes mellitus, or aging cross the blood-brain barrier. In the brain, these mediators join with pro-inflammatory mediators released by activated microglia and induce inflammation in the CNS leading to neurodegeneration. The binding of these cytokines to their receptors located in neuronal cells induces the activation of kinases such as JNK and protein kinase double-stranded RNA-dependent (PKR), which phosphorylate IRS-1 at serine residues thereby inhibiting tyrosine phosphorylation. Thus, there is blocking of the action of insulin in neuronal cells.^34^ All these factors indicate that peripheral and central inflammatory mediators contribute to neuroinflammation and resistance to neuronal insulin, which affects. among other functions, the cognitive function of diabetes and AD patients.

## CONCLUSION

Alzheimer's disease (AD) has been studied for decades. Several types of studies, including epidemiological and genetic investigations, indicate a relationship among obesity, insulin resistance, type 2 diabetes and neurodegenerative disorders such as AD.^2,76^ Some of these studies have also found a link between severe chronic inflammation and cognitive impairments in AD. Knowledge and understanding of the pathophysiology as well as inflammatory and type 2 diabetes mechanisms involved in AD are important for early diagnosis of the disease (e.g. through biomarkers) and for treatment of AD.
